# Supplementary material for: A reversible state of hypometabolism in a human cellular model of sporadic Parkinson’s disease
Source: Nat Commun. 2023 Nov 23;14:7674. doi: 10.1038/s41467-023-42862-7 (PMC10667251; doi:10.1038/s41467-023-42862-7)
Supplement: Supplementary file 1 — Supplementary Information [file 41467_2023_42862_MOESM1_ESM.pdf]

Supplementary Information File

A reversible state of hypometabolism in a human cellular model of sporadic Parkinson's disease

Authors: Sebastian Schmidt[1,2*], Constantin Stautner[1], Duc Tung Vu[3], Alexander Heinz[4], Martin Regensburger[5], Ozge Karayel[3], Dietrich Trümbach[1,6], Anna Artati[7], Sabine Kaltenhäuser[4], Mohamed Zakaria Nassef[4], Sina Hembach[1], Letyfee Steinert[1], Beate Winner[8], Jürgen Winkler[5], Martin Jastroch[9], Malte D. Luecken[10], Fabian J. Theis[10,11], Gil Gregor Westmeyer[2,12], Jerzy Adamski[13,14,15], Matthias Mann[3,16], Karsten Hiller[4], Florian Giesert[1], Daniela M. Vogt Weisenhorn[1], Wolfgang Wurst[1,17,18,19*]

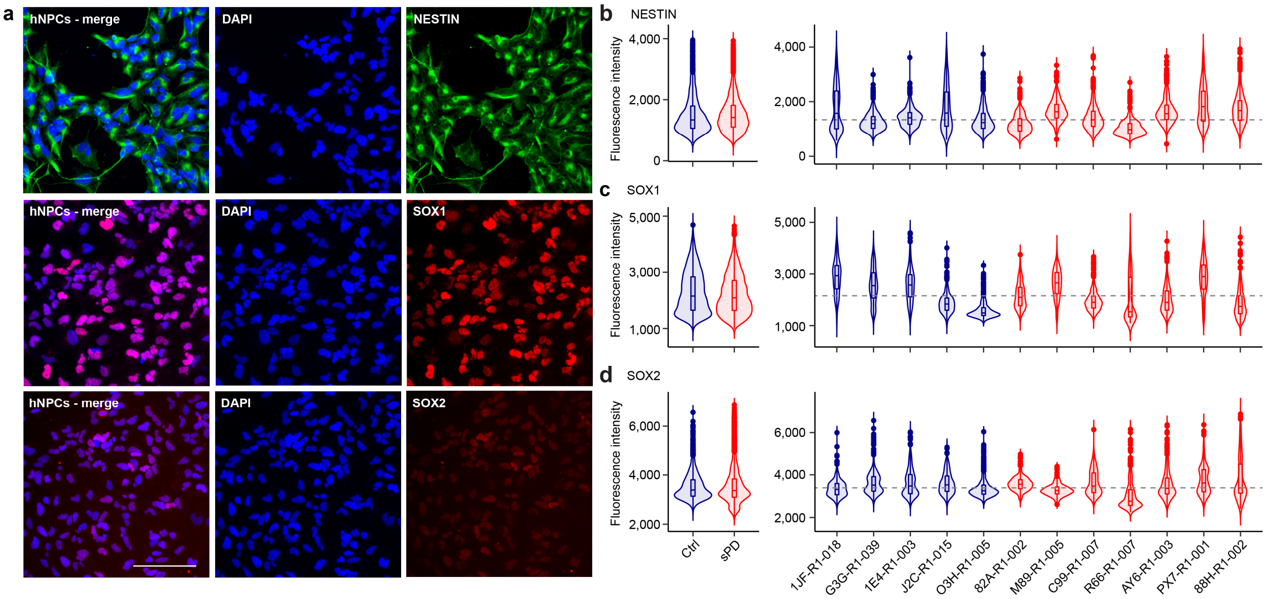

Supplementary Fig. 1 | Characterization of hiPSC-derived hNPCs. (a) Immunostainings are exemplarily shown for clone O3H-R1-003 using antibodies against the NPC markers SOX1, SOX2, NESTIN. Scale bar=100 µm. **(b)** Violin plots show the mean cytosolic fluorescence intensity of NESTIN ($p=0.94$), or **(c)** the mean nuclear fluorescence intensity of SOX1 ($p=0.57$), or **(d)** SOX2 ($p=0.95$). (left) Samples are pooled for a Ctrl-sPD comparison, or (right) values are plotted per patient-derived clone. The dashed line indicates the median fluorescence intensity level of Ctrl clones. Boxplots display the median and range from the 25th to 75th percentile. Whiskers extend to the most extreme data point which is no more than 1.5 times the interquartile range. 600 cells per clone were analyzed from n = 5 Ctrl and 7 sPD clones, in triplicates. p-values for the Ctrl-sPD comparison were determined by linear mixed effects model. *, $p < 0.05$; **, $p < 0.01$; ***, $p < 0.001$.
Source data are provided as a Source Data file.

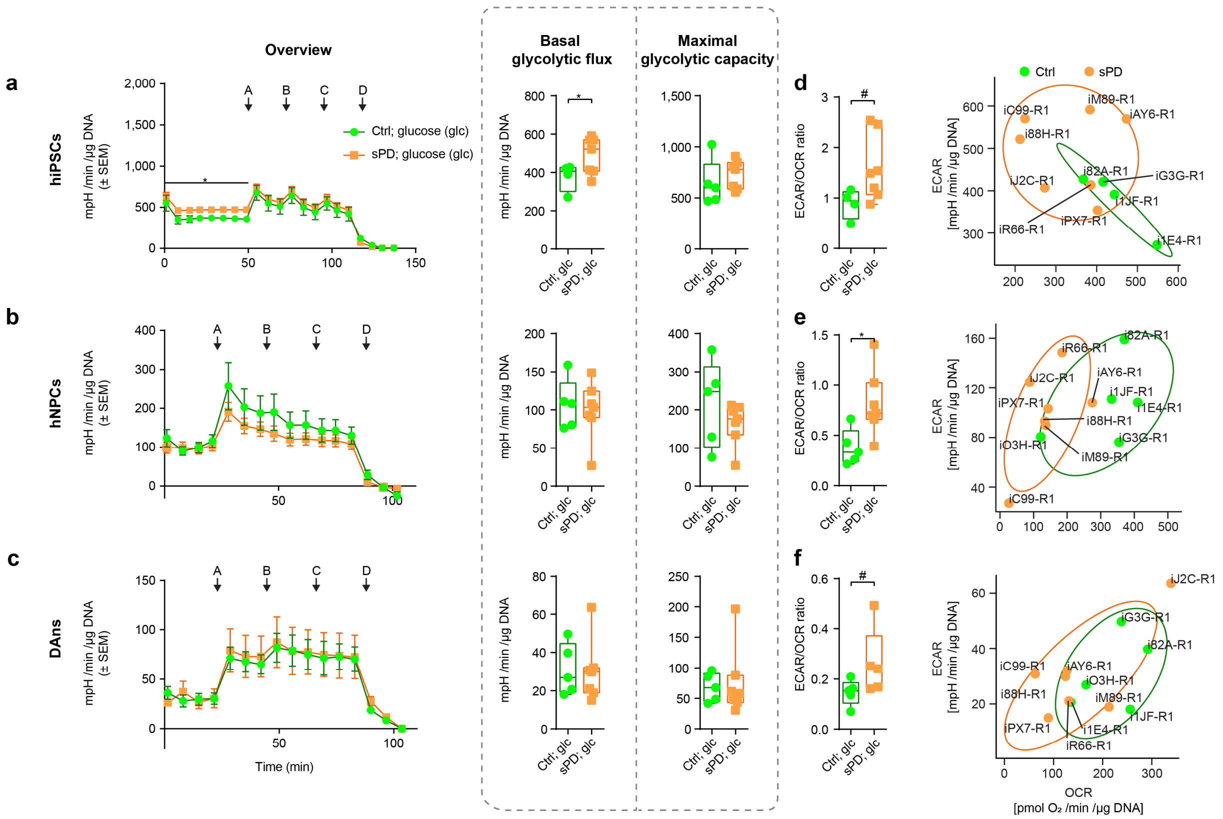

Supplementary Fig. 2 | Glycolytic flux analysis of Ctrl and sPD cell lines with glucose as energy substrate. (a) Extracellular acidification rate (ECAR) analyzed in hiPSCs, **(b)** thereof differentiated hNPCs, and **(c)** DAns using a Seahorse XFe96 Extracellular Flux Analyzer. Cells were measured in Seahorse XF assay medium supplemented with 25 mM glucose. Injected were (A) Oligomycin (1 µg/ml), (B) Carbonyl cyanide p-trifluoro-methoxyphenyl hydrazone (FCCP; 0.5 µM), (C) Rotenone (5 µM)/Antimycin A (2 µM), and (D) 2-Deoxyglucose (2-DG; 100 mM). **(d)** (left) The ECAR to OCR ratio was calculated by dividing the basal glycolytic flux by the basal mitochondrial respiration measured using glucose as energy substrate (**Fig. 1**) and reflects the cell's tendencies for ATP production. (right) The separation between Control and sPD lines is visualized by plotting the basal mitochondrial respiration (OCR) against the basal glycolytic flux (ECAR). This is shown for hiPSCs, **(e)** hNPCs, and **(f)** DAns.

Measurement progression is shown with means ± standard error of the mean (SEM). Boxplots display the median and range from the 25th to 75th percentile. Whiskers extend from the min to max value. Each dot represents one patient. n = 5 Ctrl and 7 sPD patient-derived cell clones, in triplicates. p-values were determined by two-sided *t*-test **a** (basal p=0.076; maximal: p=0.43), **b** (basal: p=0.72; maximal: p=0.31), **d** (p=0.074), **e** (p=0.022); two-sided Mann–Whitney-U test **c** (basal: p=>0.99; maximal: p=0.76), **f** (p=0.056). *, p < 0.05; **, p < 0.01; ***, p < 0.001.

Source data are provided as a Source Data file.

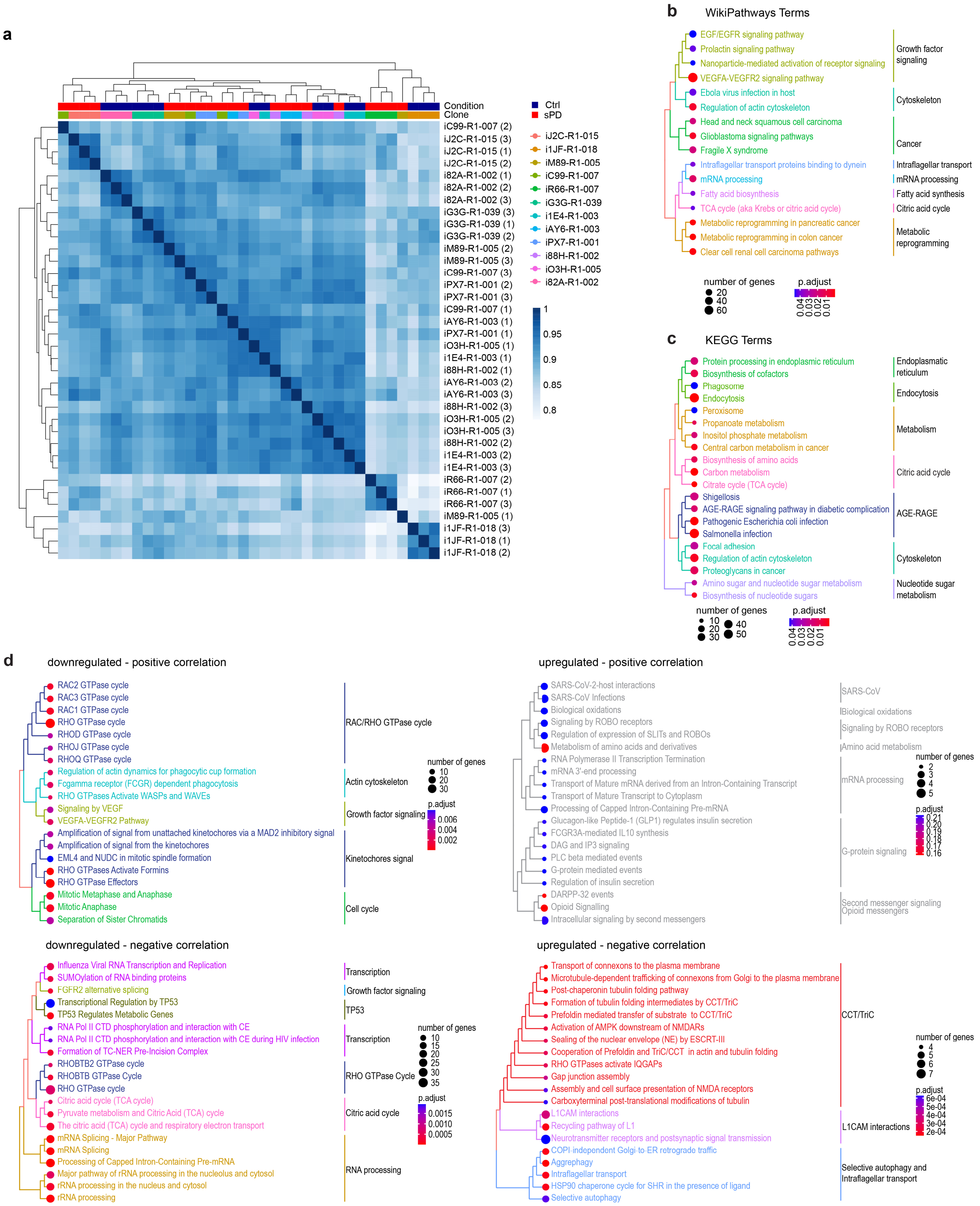

Supplementary Fig. 3 | Proteome analysis. (a) Correlation heatmap and hierarchical clustering indicating the similarity between samples. **(b)** Enriched WikiPathways and **(c)** KEGG terms for DEPs. **(d)** Enriched Reactome terms for upregulated DEGs and DEPs (top - right), downregulated DEGs and DEPs

(top - left), upregulated DEGs and downregulated DEPs (bottom - left), downregulated DEGs and upregulated DEPs (bottom - right). Not significantly enriched pathways are colored in grey.
p-values were determined by one-sided hypergeometric tests. p-values corrected for multiplicity are represented by q-values (p.adjust).

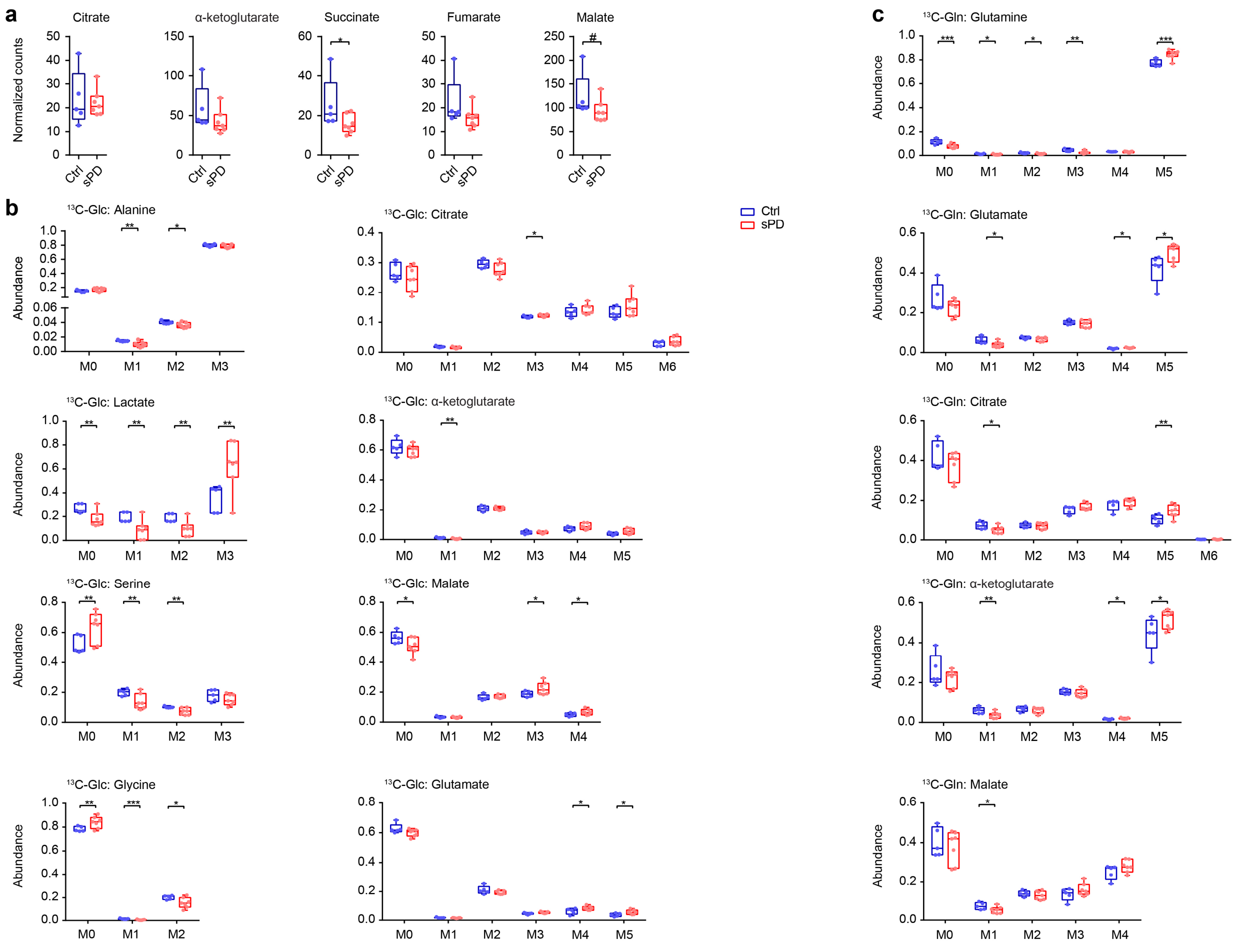

Supplementary Fig. 4 | Mass isotopomer distributions. (a) Total cellular metabolite levels quantified in [U-^{13}C]Glucose samples. **(b)** Mass isotopomer distributions (MIDs) determined by isotopic tracing with [U-^{13}C]Glucose (Glc) or **(c)** [U-^{13}C]Glutamine (Gln) and GC-MS measurement.

Boxplots display the median and range from the 25th to 75th percentile. Whiskers extend from the min to max value. Each dot represents one patient. n = 5 Ctrl and 7 sPD patient-derived cell clones, in triplicates. p-values were determined by linear mixed effects model **a** (α-ketoglutarate: p=0.15; succinate: p=0.042; malate: p=0.092; fumarate: p=0.12; citrate: p=0.72), **b**, **c**. *, p < 0.05; **, p < 0.01; ***, p < 0.001.

See also Supplementary Data 9.

Source data are provided as a Source Data file.

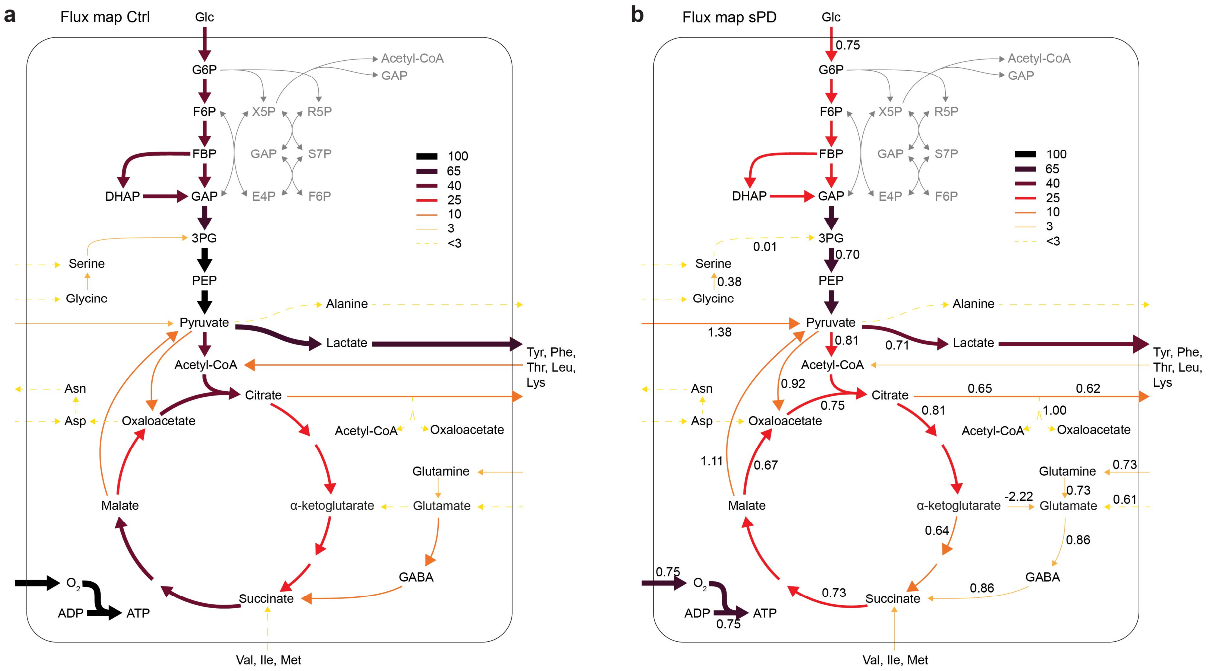

Supplementary Fig. 5 | Metabolic flux maps. (a) Metabolic flux map for Ctrl hNPCs and **(b)** sPD hNPCs. Arrow thickness and colors reflect flux values in units of mM/ h/ mg DNA. Fold changes (sPD / Ctrl hNPCs) are noted next to the respective fluxes in the map for sPD hNPCs. Fluxes colored in grey could not be determined with sufficient reliability.

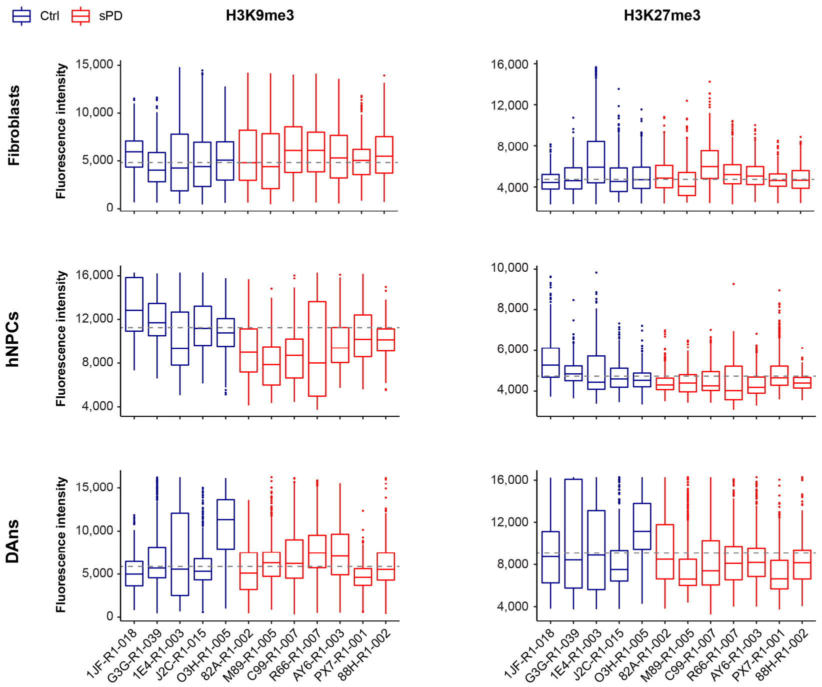

Supplementary Fig. 6 | Aging-associated histone modifications. Histone modifications H3K9me3 and H3K27me3 were analyzed in sPD and Ctrl fibroblasts as well as hNPCs and DAns derived from hiPSCs. Histone modifications were quantified from immunostainings. Dotted lines indicate the median fluorescence intensity of Ctrl clones. Boxplots display the median and range from the 25th to 75th percentile. Whiskers extend to the most extreme data point which is no more than 1.5 times the interquartile range. Experiments were performed in triplicates, n = 5 Ctrl and 7 sPD clones with 600 cells per clone.

Source data are provided as a Source Data file.

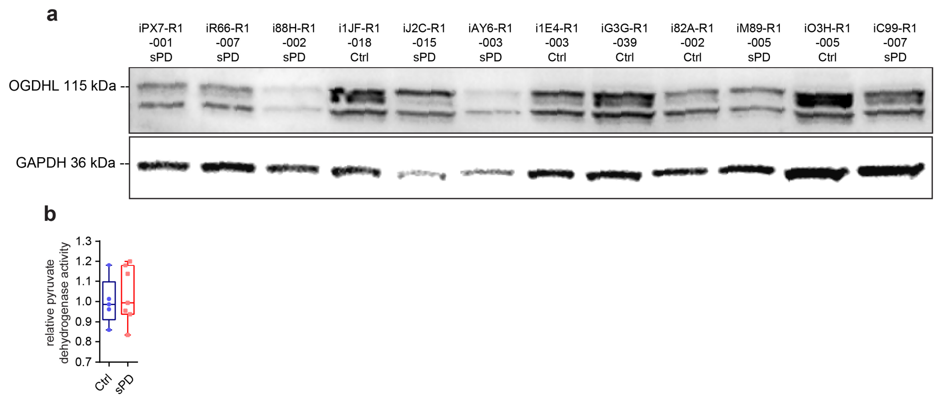

Supplementary Fig. 7 | Analysis of OGDHL abundance. The abundance of OGDHL was quantified by Western blot. Expression levels were normalized to GAPDH levels. Western blot is exemplary shown for one untreated hNPC replicate. **(b)** Activity of the pyruvate dehydrogenase complex of hNPCs. Values were normalized to mean levels of Ctrl hNPCs.

Boxplots display the median and range from the 25th to 75th percentile. Whiskers extend from the min to max value. Each dot represents one patient. n = 5 Ctrl and 7 sPD patient-derived cell clones, in triplicates. p-values were determined by two-sided *t*-test **b** (p=0.67). *, p < 0.05; **, p < 0.01; ***, p < 0.001.

Source data are provided as a Source Data file.
