# Supplementary material for: A reversible state of hypometabolism in a human cellular model of sporadic Parkinson’s disease
Source: Nat Commun. 2023 Nov 23;14:7674. doi: 10.1038/s41467-023-42862-7 (PMC10667251; doi:10.1038/s41467-023-42862-7)
Supplement: Supplementary file 3 — Description of Additional Supplementary Files [file 41467_2023_42862_MOESM3_ESM.pdf]

Description of Additional Supplementary Files

Supplementary Data 1 | Detailed description of retroviral-hiPSC clones received from the ForIPS consortium [16,17]. Column heading - study_ID_sample: identifier for hiPSC clones in this study; study_ID_individual: identifier for individuals in this study; gender: biological gender of the respective individual; age_biopsy_years: age in years of the respective individual at time of tissue biopsy; years_of_illness: time in years between sPD diagnosis and tissue biopsy of the respective individual.

Supplementary Data 2 | Transcriptome - Enriched KEGG Module terms in sPD. Enrichment analysis based on all bulk-like DEGs from sPD patients using KEGG Module terms. Enriched terms with $p < 0.05$ are shown. Column headings - ID: denotes the KEGG pathway identification number; Description: denotes the annotation term; GeneRatio: ratio of input genes that are annotated in a certain KEGG term; BgRatio: ratio of all genes that are annotated in this KEGG term to genes that are annotated in all KEGG terms; p-value: is the probability of overrepresentation calculated for each annotation term (ID) based on hypergeometric tests; p.adjust: is the p-value corrected for multiplicity by Benjamini and Hochberg; geneID: list of input genes (Entrez Gene ID) that are annotated in a certain KEGG term ; Count: number of input genes that are annotated in a certain KEGG term.

Supplementary Data 3 | Proteome analysis - DIA-MS. Detailed information regarding m/z separation and number of windows. Column headings - windows: window number used for tandem mass spectrometry analysis, m/z: the ratio of the mass (m) of an ion to its charge number (z); z: charge; RT Time: the retention time; Window (min): isolation window in minutes; Isolation Window (m/z): isolation window in m/z.

Supplementary Data 4 | Proteome - DEPs ($q < 0.05$). Column headings – UniProtIds; GeneSymbol: official HGNC gene symbol; baseMean: Mean of normalized counts; log2FoldChange: fold change of DEPs in sPD; lfcSE: standard error estimate for the log2 fold change; stat: Wald statistic; pvalue: Wald test p-value; padj: p-value corrected for multiplicity.

Supplementary Data 5 | Proteome - Enriched pathways in sPD. Enrichment analysis based on all DEPs from sPD patients using KEGG, KEGG Module, WikiPathway, and Reactome terms. Enriched terms with $p < 0.05$ are shown. Column heading - ID: denotes the pathway identification number; Description: denotes the annotation term; GeneRatio: ratio of input genes that are annotated in a certain pathway; BgRatio: ratio of all genes that are annotated in this pathway to genes that are annotated in all pathways; p-value: is the probability of overrepresentation calculated for each annotation term (ID) based on hypergeometric tests; p.adjust: is the p-value corrected for multiplicity by Benjamini and Hochberg; geneID: list of input genes (Entrez Gene ID) that are annotated in a certain pathway; Count: number of input genes that are annotated in a certain pathway.

Supplementary Data 6 | Proteome - Enriched pathways of correlating DEG and DEPs in sPD. Enrichment analysis based on DEG-DEP pairs from sPD patients with a positive or negative correlation using Reactome terms. Enriched terms with $p < 0.05$ are shown. Column headings - ID: denotes the pathway identification number; Description: denotes the annotation term; GeneRatio: ratio of input genes that are annotated in a certain pathway; BgRatio: ratio of all genes that are annotated in this pathway to genes that are annotated in all pathways; p-value: is the probability of overrepresentation calculated for each annotation term (ID) based on hypergeometric tests; p.adjust: is the p-value corrected for multiplicity by Benjamini and Hochberg; geneID: list of input genes (Entrez Gene ID) that are annotated in a certain pathway; Count: number of input genes that are annotated in a certain pathway.

Supplementary Data 7 | Non-targeted metabolome - significantly altered metabolites ($q < 0.05$). Column headings - CHEMICAL: internal identification number; mean Ctrl: Mean of normalized counts of Ctrl samples; mean sPD: Mean of normalized counts of sPD samples; fc: fold change of metabolites in sPD; p-value shapiro-test: p-value calculated by Shapiro-Wilks test for normality; p-value wilcox-test: p-value calculated by two-sided Wilcoxon-tests; p.adj wilcox-test: FDR-corrected p-value; p-value t-test: p-value calculated by two-sided t-tests; p.adj t-test: FDR-corrected p-value; BIOCHEMICAL: Biochemical nomenclature; SUB PATHWAY: Metabolite is assigned to these subsets of biochemical pathways; SUPER PATHWAY: Metabolite is assigned to these biochemical pathways; PUBCHEM: identification number; KEGG: identification number; HMDB: identification number.

Supplementary Data 8 | Non-targeted metabolome - Integrated pathway enrichment analysis. Enrichment analysis based on all DEPs or bulk-like DEGs and significantly altered metabolites from sPD patients using MetaboAnalyst and metabolic KEGG terms. Column headings - Description: KEGG pathway description; Total: Total number of compounds in pathways; Expected: Expected number of hits; Hits: Total number of hits; Raw p: is the probability of overrepresentation calculated for each annotation term based on hypergeometric tests; -LOG10(p): $-\log_{10}$ transformed p-values; Holm adjust: p-value corrected for multiplicity by Holm-Bonferroni; FDR: FDR-corrected p-value; Impact: Calculated impact of hits on pathways based on network topology analysis.

Supplementary Data 9 | ^{13}C labeling - Mass isotopomer distributions. MIDs were yielded from [U-^{13}C]Glucose and [U-^{13}C]Glutamine labeling experiments. Column headings - Metabolite: Combination of biochemical nomenclature_mass-to-charge-ratio_number of incorporated ^{13}C; following column names e.g. i1JF-R1-018_1 indicate a unique sample name as a combination of cell line identification number_number of technical replicates. These columns contain the corrected mass isotopomer distributions for labeled metabolites. Distributions per metabolite add up to 1.

Supplementary Data 10 | Metabolic flux analysis. Metabolic flux maps for Ctrl and sPD hNPCs based on mass isotopomer distributions and extracellular uptake/secretion rates. Column headings - Reaction_ID: Internal reaction ID allows to discriminate between reactions and their net or exchange (exch) fluxes; Reaction_Equation: Equations specified in the metabolic network; Flux_value: Optimal estimated flux value; Flux_StdErr: Estimated standard error of fluxes; Flux_LB: Computed lower bound of the 95% confidence interval; Flux_UB: Computed upper bound of the 95% confidence interval; Fold change: fold change of fluxes in sPD.
