# Supplementary material for: A reversible state of hypometabolism in a human cellular model of sporadic Parkinson’s disease
Source: Nat Commun. 2023 Nov 23;14:7674. doi: 10.1038/s41467-023-42862-7 (PMC10667251; doi:10.1038/s41467-023-42862-7)
Supplement: Supplementary file 14 — Reporting Summary [file 41467_2023_42862_MOESM14_ESM.pdf]

nature portfolio

Corresponding author(s): Wolfgang Wurst, Sebastian Schmidt

Last updated by author(s): Oct 11, 2023

Reporting Summary

Nature Portfolio wishes to improve the reproducibility of the work that we publish. This form provides structure for consistency and transparency in reporting. For further information on Nature Portfolio policies, see our Editorial Policies and the Editorial Policy Checklist.

Statistics

For all statistical analyses, confirm that the following items are present in the figure legend, table legend, main text, or Methods section.

n/a	Confirmed	
☐	☒	The exact sample size (*n*) for each experimental group/condition, given as a discrete number and unit of measurement
☐	☒	A statement on whether measurements were taken from distinct samples or whether the same sample was measured repeatedly
☐	☒	The statistical test(s) used AND whether they are one- or two-sided<br>*Only common tests should be described solely by name; describe more complex techniques in the Methods section.*
☐	☒	A description of all covariates tested
☐	☒	A description of any assumptions or corrections, such as tests of normality and adjustment for multiple comparisons
☐	☒	A full description of the statistical parameters including central tendency (e.g. means) or other basic estimates (e.g. regression coefficient) AND variation (e.g. standard deviation) or associated estimates of uncertainty (e.g. confidence intervals)
☐	☒	For null hypothesis testing, the test statistic (e.g. *F*, *t*, *r*) with confidence intervals, effect sizes, degrees of freedom and *P* value noted<br>*Give P values as exact values whenever suitable.*
☒	☐	For Bayesian analysis, information on the choice of priors and Markov chain Monte Carlo settings
☒	☐	For hierarchical and complex designs, identification of the appropriate level for tests and full reporting of outcomes
☐	☒	Estimates of effect sizes (e.g. Cohen's *d*, Pearson's *r*), indicating how they were calculated

Our web collection on statistics for biologists contains articles on many of the points above.

Software and code

Policy information about availability of computer code

Data collection

No software was used for data collection.

Data analysis

Running in R version 4.2.2 and RStudio 2022.12.0 Build 353 (if not stated otherwise) :
For DEP analysis, hypothesis testing was performed using the package DESeq2 v 1.36.0.
Enriched gene sets were analyzed using the R package clusterProfiler version 4.2.2 or ReactomePA v1.38.0.
Pathways (source: WikiPathways) were visualized and annotated using cytoscape v3.9.1 (installed apps: WikiPathways v3.3.10, CyTargetLinker v4.1.0, stringApp v1.7.0, and enhancedGraphics v1.5.4) and the R package RCy3 v2.14.2.
Heatmaps were generated by using the R package gplots v3.1.1.
Vulcano plots were generated by using the R package EnhancedVolcano version 1.12.0.
Distribution plots were generated and compared using the R package “sm: Smoothing Methods for Nonparametric Regression and Density Estimation” version 2.2-5.681.
Venn diagrams were generated using the R package “VennDiagram” Version 1.6.20.
Linear mixed effects model were fit using the lmer function (R package “lme4” v1.1-31), p values were calculated using the Anova function (R package “car” Version 3.0-10).
p-values were adjusted for multiple testing using the R package fdrtool v1.2.15 (statistic = “pvalue”, cutoff.method = “ptc0”).
PCA was performed using the prcomp function in R and visualized using the R package ggbiplot v0.55.
Correlation plots were generated using ggplot2 v3.4.0.
Correlation heatmaps were plotted using the pheatmap function of the R package pheatmap v1.0.12.
The multiple factor analysis was performed using the R package FactorMineR v2.8. The multiple factor analysis was visualized using the R package factoextra v1.0.7.
The Pearson correlation between parameters for the whole dataset or the sPD dataset was performed using the function rcorr of the R

package Hmisc v5.1-0.
A correlogram showing the Pearson correlation coefficients was generated using the function corrplot of the R package corrplot v0.92.
Scatter plots were generated using the function ggscatter of the R package ggpubr v0.6.0. Pearson correlation coefficients with p-values were added using the function stat_cor.
Violin plots were generated using the functions ggplot + geom_violin of the R package ggplot2 v3.4.2.
Boxplots summarizing measurements per individual cell line were generated using the functions ggplot + geom_boxplot of the R package ggplot2 v3.4.2.

Running in other programs:
All other visualizations and statistical analyses were performed using GraphPad Prism 6.
Proteins were filtered and imputed using Perseus version 1.6.1.3.
ImageJ 1.53c was used for image processing.
Image Lab 6.1 was used for western blot quantification.
High content screening was performed using HCS Studio 2.0.
Wave 2.6.1 was used for visualizing and exporting Seahorse XF data.
DIA raw files were analyzed using directDIA in Spectronaut version 15 (Biognosys).
Non-stationary 13C-MFA was performed using INCA v2.1 40,69 within MATLAB R2018a (installed packages: Statistics and Optimization Toolbox).
Transcription factor binding sites were predicted using JASPAR, release 9 (2022).
Integrated pathway enrichment analysis of transcriptome or proteome data and metabolome data was performed in MetaboAnalyst v5.0.
Quantification of mitochondria or glucose transporter was performed using CellProfiler v4.2.5.
For non-targeted metabolomics, analysis was performed using Metabolon's hardware and software (Metabolon, Inc., North Carolina, USA).
For isotopic labeling, The MetaboliteDetector software was used to analyze chromatograms, calculate mass isotopomer distributions (MIDs) and perform relative comparison of metabolite levels.
JASPAR, release 9 (2022) was used for predicting transcription factor bonding sites.

Analysis code is freely available a [https://github.com/sebischmidt/A-reversible-state-of-hypometabolism-in-a-cellular-model-of-sporadic-Parkinson-s-disease].

For manuscripts utilizing custom algorithms or software that are central to the research but not yet described in published literature, software must be made available to editors and reviewers. We strongly encourage code deposition in a community repository (e.g. GitHub). See the Nature Portfolio guidelines for submitting code & software for further information.

Data

Policy information about availability of data

All manuscripts must include a data availability statement. This statement should provide the following information, where applicable:

- Accession codes, unique identifiers, or web links for publicly available datasets
- A description of any restrictions on data availability
- For clinical datasets or third party data, please ensure that the statement adheres to our policy

All data produced in this study are archived internally. Further information and requests for resources and reagents should be directed to and will be fulfilled by the corresponding authors. Source data are provided with this paper.
The mass spectrometry proteomics data have been deposited to the ProteomeXchange Consortium via the PRIDE partner repository with the dataset identifier PXD038399 [http://www.ebi.ac.uk/pride/archive/projects/PXD038399].
13C labeling data have been deposited in the Metabolomics Workbench under project ID PR001536 and study ID ST002389 79 [https://www.metabolomicsworkbench.org/data/DRCCMetadata.php?Mode=Study&StudyID=ST002389&StudyType=MS&ResultType=1].
Following databases were used within this study: KEGG pathways [https://www.genome.jp/kegg/pathway.html] (downloaded on 11.2022), WikiPathway pathways [https://www.wikipathways.org/index.php/WikiPathways] (downloaded on 11.2022), Reactome pathways [https://reactome.org/] (downloaded on 11.2022), Genomatix ElDorado database version 04-2021 (genome build GRCh38) [https://mygga.genomatix.de/online_help//help_eldorado/GenomeBrowser.html] (downloaded on 04.2021), UCSC genome browser [https://genome.ucsc.edu/] (accessed 11.2022), KEGG pathways used with MetaboAnalyst v5.0 [https://www.metaboanalyst.ca/MetaboAnalyst/home.xhtml] (downloaded 10.2019).

Research involving human participants, their data, or biological material

Policy information about studies with human participants or human data. See also policy information about sex, gender (identity/presentation), and sexual orientation and race, ethnicity and racism.

Reporting on sex and gender	Individuals were recruited independently of their sex and gender. The sex and gender was determined based on self-report. Sex or gender differences were not considered for the study design. Data have not been aggregated or analyzed individually for different sex/genders. Findings apply to both male and female.
Reporting on race, ethnicity, or other socially relevant groupings	We did not consider data about or group individuals based on race, ethnicity, or other socially relevant categories. Grouping was only performed based on the medical condition as described below.
Population characteristics	See also Table 1.<br>Disease progression in sPD patients within 10 years after biopsy. (left) Description of sPD patients at the timepoint of skin biopsy (Gender, and time in years between sPD diagnosis and tissue biopsy). (right) Long term history of sPD patients. Changes in the Hoehn&Yahr scale (Δ H&Y), in motor examinations (Part III) according to the Unified Parkinson Disease Rating

	Scale (Δ UPDRS III), medication requirement (Δ L-Dopa equivalent), and "activities of daily living" (Δ ADL) monitored within 9-12 years after skin biopsy.<br>Patient ID; Gender; Years of illness; Δ H&Y; Δ UPDRS III [points]; Δ L-Dopa equivalent [mg]; Δ ADL<br>J2C; m; 3; 3; 24; 400; -0.6<br>M89; m; 3; 3; 53; 1563; -0.6<br>C99; m; 7; 2.5; 36; 610; -0.5<br>R66; m; 3; Follow-up not available<br>AY6; m; 4; 1; -1; 800; -0.2<br>PX7; m; 1; 1; 0; 1900; -0.1<br>88H; f; 6; 2; 7; 640; -0.3
Recruitment	Individuals were recruited independently of their sex/gender, race, ethnicity, or other socially relevant categories.<br>Participants were not compensated.<br>Selection criteria were solely based on the medical condition:<br>Individuals were phenotypically examined and grouped by a clinician experienced with neurological diseases.<br>Individuals without any neurologic disease were included as controls.<br>Both at baseline and at follow-up, all PD patients fulfilled the clinical diagnostic criteria for PD (Ref53) and were defined as having sPD by the absence of known PD-causing familial mutations (PARK 1-18) and a negative family history of PD (Ref54).<br>Disease progression was assessed by monitoring changes in PD-associated scores determined according to the Hoehn & Yahr scale (H&Y), part III (motor symptoms) of the Unified Parkinson's Disease Rating Scale (UPDRS), as well as the activities of daily living (ADL) scale.<br><br>53. Postuma, R. B. et al. MDS clinical diagnostic criteria for Parkinson's disease. Movement disorders : official journal of the Movement Disorder Society 30, 1591–1601; 10.1002/mds.26424 (2015).<br>54. Popp, B. et al. Need for high-resolution Genetic Analysis in iPSC: Results and Lessons from the ForIPS Consortium. Scientific reports 8, 17201; 10.1038/s41598-018-35506-0 (2018).
Ethics oversight	The use of patient related material, information, and cell culture work was approved by local ethics committees (No. 4485, No. 4120, No. 17-259, FAU Erlangen-Nuernberg, Germany; and No 422-13 and 357/19 S, Technical University Munich, Germany) and all participants or their legal guardians gave written informed consent. All related examinations, experiments and methods were performed in accordance with relevant guidelines and regulations.

Note that full information on the approval of the study protocol must also be provided in the manuscript.

Field-specific reporting

Please select the one below that is the best fit for your research. If you are not sure, read the appropriate sections before making your selection.

☒ Life sciences ☐ Behavioural & social sciences ☐ Ecological, evolutionary & environmental sciences

For a reference copy of the document with all sections, see nature.com/documents/nr-reporting-summary-flat.pdf

Life sciences study design

All studies must disclose on these points even when the disclosure is negative.

Sample size	No explicit calculations were performed to determine sample size since all (at this time) available and well characterized hiPSC lines provided by the ForIPS consortium were used for analysis. Thus, we used the largest available sample size possible. The sample size was sufficient to perform reliable data analysis.
Data exclusions	Metabolomics: Only metabolites or MIDs that were detected in more than 30% of all replicates were used for statistical analysis. Remaining metabolites/MIDs were excluded.<br>Proteomics: Two human-derived cell lines (sPD - R66-R1-007 and Ctrl - 1JF-R1-018) seemed to cluster separately and thus were removed as outliers for downstream analysis.<br>For all other experiments, outliers identified by the ROUT method (Q = 0.5%; GraphPad) were removed for statistical analysis
Replication	Proteomics: Three replicates per cell clone were collected.<br>Non-targeted Metabolomics: Five replicates per cell clone were collected.<br>Isotopic labeling: Three replicates per cell clone were collected.<br>For all remaining experiments, mean values from three independent repetitions per cell line are shown and were used for statistical analysis. All experiments were successfully repeated three times. This takes not into account the repetitions that had to be discarded due to technical issues (e.g. detection problems of bands on western<br>blots).
Randomization	Individuals were phenotypically examined and grouped by a clinician experienced with neurological diseases.<br>Individuals without any neurologic disease were included as controls.<br>Both at baseline and at follow-up, all PD patients fulfilled the clinical diagnostic criteria for PD (Ref53) and were defined as having sPD by the absence of known PD-causing familial mutations (PARK 1-18) and a negative family history of PD (Ref54). Disease progression was assessed by monitoring changes in PD-associated scores determined according to the Hoehn & Yahr scale (H&Y), part III (motor symptoms) of the Unified Parkinson's Disease Rating Scale (UPDRS), as well as the activities of daily living (ADL) scale.<br><br>53. Postuma, R. B. et al. MDS clinical diagnostic criteria for Parkinson's disease. Movement disorders : official journal of the<br>Movement Disorder Society 30, 1591–1601; 10.1002/mds.26424 (2015).

54. Popp, B. et al. Need for high-resolution Genetic Analysis in iPSC: Results and Lessons from the ForIPS Consortium. Scientific reports 8, 17201; 10.1038/s41598-018-35506-0 (2018).

Blinding

Investigators were blinded to group allocation for data collection, processing and exclusions.

Reporting for specific materials, systems and methods

We require information from authors about some types of materials, experimental systems and methods used in many studies. Here, indicate whether each material, system or method listed is relevant to your study. If you are not sure if a list item applies to your research, read the appropriate section before selecting a response.

Materials & experimental systems

n/a	Involved in the study
☐	☒ Antibodies
☐	☒ Eukaryotic cell lines
☒	☐ Palaeontology and archaeology
☒	☐ Animals and other organisms
☒	☐ Clinical data
☒	☐ Dual use research of concern
☒	☐ Plants

Methods

n/a	Involved in the study
☒	☐ ChIP-seq
☒	☐ Flow cytometry
☒	☐ MRI-based neuroimaging

Antibodies

Antibodies used

Primary antibodies Western Blot:
NDUFB8 (459210, Novex; 1:500),
Complex II- Subunit 30 (459230, Thermo Fisher Scientific; 1:500),
UQCRC2 (ab14745, Abcam; 1:2,500),
MTCO2 (ab110258, Abcam; 1:1,000),
ATP5F1A (ab14748, Abcam; 1:4,000),
DRP1 (5391, Cell Signaling; 1:1,000),
DRP1 phospho-Ser616 (4494, Cell Signaling; 1:1,000),
TUBA (GTX628802, Genetex; 1:20,000),
ACTB (ABO145-200, OriGene; 1:2,000) ,
OGDHL (17110-1-AP, Proteintech, 1:5,000),
GAPDH (GTX627408 peroxidase coupled, Genetex, 1:20,000),
SLC2A1 (MA5-31960, Invitrogen, 1:500),
SLC2A3 (PA5-72331, Thermo Fisher Scientific, 1:500)

Primary antibodies immunostainings:
ATP5F1A (ab14748, Abcam; 1:500),
NES (Ma1110, Thermo Fisher Scientific; 1:250),
Histone H3 (tri methyl K27) (ab6002, Abcam; 1:100),
Histone H3 (tri methyl K9) (ab8898, Abcam; 1:500),
SLC2A1 (MA5-31960, Invitrogen; 1:500),
SLC2A3 (PA5-72331, Thermo Fisher Scientific; 1:500),
SOX1 (Ab87775, Abcam; 1:500),
SOX2 (sc17320, Santa Cruz; 1:500),
TUBB3 (T5076, Sigma-Aldrich; 1:1000)

Secondary antibodies:
rabbit-anti-mouse IgG peroxidase (GTX213112-01, GeneTex; 1:10,000),
goat-anti-rabbit IgG peroxidase (111-035-003, Dianova; 1:10,000),
donkey-anti-rabbit IgG Alexa 488 (A21206, Thermo Fisher Scientific; 1:500)
donkey-anti-goat IgG Alexa 488 (A11055, Thermo Fisher Scientific; 1:500),
donkey-anti-goat IgG Alexa 594 (A11058, Thermo Fisher Scientific; 1:500),
donkey-anti-mouse IgG Alexa 594 (A21203, Thermo Fisher Scientific; 1:500),
donkey-anti-rabbit IgG Alexa 594 (A21207, Thermo Fisher Scientific; 1:500),
rabbit-anti-goat IgG peroxidase (305-035-003, Dianova; 1:10,000)
donkey-anti-mouse IgG Alexa 488 (A21202, Thermo Fisher Scientific; 1:500),

Validation

NDUFB8 (Novex; 459210; 1:500 ; applications: Immunohistochemistry, Immunohistochemistry-paraffin, Western Blotting; reactivity: Bovine, Human, House mouse, Rat; validation: 33x -e.g. Palermo et al., 2022; Basse et al. 2021),
Complex II- Subunit 30 (Thermo Fisher Scientific; 459230; 1:500; applications: Flow cytometry, Immunofluorescence, Immunohistochemistry, Western Blotting; reactivity: Bovine, Human, House mouse, Rat; validation: 15x- e.g. Deitersen et al., 2021; Cao et al., 2020),
UQCRC2 (Abcam; ab14745; 1:2,500; applications: Flow cytometry/Cell sorting, Immunohistochemistry, Immunohistochemistry-paraffin, Western Blotting; reactivity: Bovine, Human, House mouse, Rat, Pig; validation: 301x - e.g. Chandler et al., 2023; Chen et al., 2023),
MTCO2 (Abcam; ab110258; 1:1,000; applications: Flow cytometry/Cell sorting, Immunofluorescence, Immunohistochemistry,

Immunohistochemistry-paraffin, Western Blotting; reactivity: Human; validation: 242x- e.g. Budzinska et al., 2023; Russo et al., 2023),
ATP5F1A (Abcam; ab14748; 1:4,000; applications: Flow cytometry/Cell sorting, Immunocytochemistry, Immunofluorescence, Immunohistochemistry-paraffin, Immunoprecipitation, Western Blotting; reactivity: Bovine, Caenorhabditis elegans, Green monkey, Drosophila melanogaster, Human, House mouse, Rat, Pig; validation: 598x- e.g. Serrano-Lorenzo et al., 2023; Papadaki et al., 2023),
DRP1 (Cell Signaling; 5391; 1:1,000; applications: Immunoprecipitation, Western Blotting; reactivity: Green monkey, Human, House mouse, Rat; validation: 74x- e.g. Rios et al., 2023; Moon et al., 2023),
DRP1 phospho-Ser616 (Cell Signaling; 4494; 1:1,000; applications: Flow cytometry/Cell sorting, Immunofluorescence, Western Blotting; reactivity: Human; validation: 130x- e.g. Wang et al., 2023; Li et al., 2023),
TUBA (Genetex; GTX628802; 1:20,000; applications: Flow cytometry/Cell sorting, Immunocytochemistry, Immunofluorescence, Immunohistochemistry, Immunohistochemistry-paraffin, Western Blotting; reactivity: Danio rerio, Drosophila melanogaster, Human, House mouse, Rat; validation: 142x- e.g. Niesen et al., 2023; Ozgencil et al., 2023),
ACTB (OriGene; ABO145-200; 1:2,000; applications: Immunofluorescence, Western Blotting; reactivity: Canine, Human, Monkey, House mouse, Rat; validation: 1x- Ishizuka et al., 2020),
OGDHL (Proteintech; 17110-1-AP; 1:5,000; applications: Enzyme-linked immunosorbent assay, Immunohistochemistry, Immunoprecipitation, Western Blotting; reactivity: Human, House mouse, Rat; validation: 11x- e.g. Jiang et al., 2023; Xu et al.; 2023),
GAPDH (Genetex; GTX627408 peroxidase coupled; 1:20,000; applications: Electrophoretic mobility shift assay, Immunocytochemistry, Immunofluorescence, Immunohistochemistry-paraffin, Western Blotting; reactivity: Candida albicans, Green monkey, Danio rerio, Drosophila melanogaster, Escherichia coli, Human, House mouse, Saccharomyces cerevisiae, Pig; validation: 426x- e.g. Meng et al., 2023; Liang et al., 2023),
SLC2A1 (Invitrogen; MA5-31960; 1:500 ; applications: Flow cytometry/Cell sorting, Immunocytochemistry, Immunofluorescence, Immunohistochemistry, Western Blotting; reactivity: Human, House mouse, Rat; validation: 10x- e.g. Burgio et al., 2023; Zhang et al., 2023),
SLC2A3 (Thermo Fisher Scientific; PA5-72331; 1:500; applications: Western Blotting; reactivity: Human, House mouse, Rat; validation: 1x- Mamun et al., 2020),

ATP5F1A (Abcam; ab14748; 1:500; applications: Flow cytometry/Cell sorting, Immunocytochemistry, Immunofluorescence, Immunohistochemistry-paraffin, Immunoprecipitation, Western Blotting; reactivity: Bovine, Caenorhabditis elegans, Green monkey, Drosophila melanogaster, Human, House mouse, Rat, Pig; validation: 598x- e.g. Serrano-Lorenz et al., 2023; Papadaki et al., 2023),
NES (Thermo Fisher Scientific; Ma1110; 1:250; applications: Flow cytometry, Immunofluorescence, Immunohistochemistry, Immunohistochemistry-frozen, Immunohistochemistry-paraffin, Western Blotting; reactivity: Human, House mouse; validation: 33x- e.g. Jiang et al., 2023; Kim et al., 2023),
Histone H3 (tri methyl K27) (Abcam; ab6002; 1:100; applications: Chromatin immunoprecipitation, Enzyme-linked immunosorbent assay, Flow cytometry/Cell sorting, Immunocytochemistry, Immunofluorescence, Immunohistochemistry-paraffin; reactivity: Arabidopsis thaliana, Bovine, Cricetulus griseus, Danio rerio, Drosophila melanogaster, Chicken, Human, Rhesus Monkey; validation: 1,037x- e.g. Izzo et al., 2023; Chen et al., 2023),
Histone H3 (tri methyl K9) (Abcam; ab8898; 1:500; applications: Chromatin immunoprecipitation, Flow cytometry/Cell sorting, Immunofluorescence, Immunohistochemistry, Immunohistochemistry-paraffin, Western Blotting; reactivity: Cyanidioschyzon merolae, Drosophila melanogaster, Chicken, Human, Mammalia, Muntiacus muntjak, House mouse, Rat; validation: 1,851x- e.g. Kiseleva et al., 2023; Ihashi et al., 2023),
SLC2A1 (Invitrogen; MA5-31960; 1:500; applications: Flow cytometry/Cell sorting, Immunocytochemistry, Immunofluorescence, Immunohistochemistry, Western Blotting; reactivity: Human, House mouse, Rat; validation: 10x- e.g. Burgio et al., 2023; Zhang et al., 2023),
SLC2A3 (Thermo Fisher Scientific; PA5-72331; 1:500; applications: Western Blotting; reactivity: Human, House mouse, Rat; validation: 1x- Mamun et al., 2020),
SOX1 (Abcam; Ab87775; 1:500; applications: Immunocytochemistry-immunofluorescence, Immunohistochemistry-paraffin, Western Blotting; reactivity: Domestic dog, Human, House mouse, Rabbit, Common chimpanzee, Orangutan, Rat; validation: 26x- e.g. Lebedeva et al., 2023; Ben-Zvi et al., 2022),
SOX2 (Santa Cruz; sc17320; 1:500; applications: Immunofluorescence, mmunohistochemistry-paraffin, Western Blotting; reactivity: Human, House mouse, Rat; validation: 44x- e.g. Huang et al., 2023; Karapurkar et al., 2023),
TUBB3 (Sigma-Aldrich; T5076; 1:1,000; applications: Antibody array, Dot blot, Enzyme-linked immunosorbent assay, Immunocytochemistry, Immunohistochemistry, Western Blotting; reactivity: Bovine, Human, House mouse, Rat, Pig; validation: 90x- e.g. Kandhavivorn et al., 2023; Pedrosa et al.; 2023),

rabbit-anti-mouse IgG peroxidase (GeneTex; GTX213112-01; 1:10,000; applications: Enzyme-linked immunosorbent assay, Western Blotting; reactivity: House mouse; validation: 21x- e.g. Kim et al., 2023; Lin et al., 2023),
goat-anti-rabbit IgG peroxidase (Dianova; 111-035-003; 1:10,000; applications: Enzyme-linked immunosorbent assay, Immunohistochemistry, Western Blotting; reactivity: Rabbit; validation: 2,323x- e.g. Shima et al., 2023; Bosnakovski et al., 2023),
rabbit-anti-goat IgG peroxidase (Dianova; 305-035-003; 1:10,000; applications: Enzyme-linked immunosorbent assay, Immunohistochemistry, Western Blotting; reactivity: Goat; validation: 99x- e.g. Vlk et al., 2023; de Souza et al., 2023),
donkey-anti-mouse IgG Alexa 488 (Thermo Fisher Scientific; A21202; 1:500; applications: Immunocytochemistry, Immunofluorescence, Immunohistochemistry; reactivity: House mouse; validation: 4,527x- e.g. Shi et al., 2023; Tanday et al., 2023),
donkey-anti-rabbit IgG Alexa 488 (Thermo Fisher Scientific; A21206; 1:500; applications: Flow cytometry/Cell sorting, Immunocytochemistry, Immunofluorescence, Immunohistochemistry; reactivity: Rabbit; validation: 6,182x- e.g. Shorthouse et al., 2023; Pantalone et al., 2023),
donkey-anti-goat IgG Alexa 488 (Thermo Fisher Scientific; A11055; 1:500; applications: Flow cytometry/Cell sorting, Immunocytochemistry, Immunofluorescence, Immunohistochemistry; reactivity: Goat; validation: 3,002x- e.g. Hashida et al., 2023; Tanday et al., 2023),
donkey-anti-goat IgG Alexa 594 (Thermo Fisher Scientific; A11058; 1:500; applications: Flow cytometry/Cell sorting, Immunocytochemistry, Immunofluorescence, Immunohistochemistry; reactivity: Goat; validation: 1,044x- e.g. Alfano et al., 2023; Furue et al., 2023),
donkey-anti-mouse IgG Alexa 594 (Thermo Fisher Scientific; A21203; 1:500; applications: Immunocytochemistry, Immunofluorescence, Immunohistochemistry; reactivity: House mouse; validation: 1,453x- e.g. Pellegrino et al., 2023; Cheng et al., 2023),
donkey-anti-rabbit IgG Alexa 594 (Thermo Fisher Scientific; A21207; 1:500; applications: Flow cytometry/Cell sorting,

Immunocytochemistry, Immunofluorescence, Immunohistochemistry; reactivity: Rabbit; validation: 2,304x- e.g. Shorthouse et al. 2023; Ikeda et al., 2023)

Eukaryotic cell lines

Policy information about cell lines and Sex and Gender in Research

Cell line source(s)	sPD and Ctrl hiPSC lines were established, characterized and provided by the ForIPS consortium (University Hospital Erlangen) (Supplementary Data 1).
Authentication	Cell lines were authenticatedin the ForIPS consortium by analyzing 20 polymorphic markers located on 15 different chromosomes to exclude handling errors. This has been recently published by Popp et al. 2018 [https://doi.org/10.1038/s41598-018-35506-0].<br>At the end of experiments, karyotyping was repeated to ensure the integrity of all cell lines.
Mycoplasma contamination	All cell lines were tested negative for mycoplasma contamination.
Commonly misidentified lines<br>(See ICLAC register)	No misidentified cell line was used.

Plants

Seed stocks	*Report on the source of all seed stocks or other plant material used. If applicable, state the seed stock centre and catalogue number. If plant specimens were collected from the field, describe the collection location, date and sampling procedures.*
Novel plant genotypes	*Describe the methods by which all novel plant genotypes were produced. This includes those generated by transgenic approaches, gene editing, chemical/radiation-based mutagenesis and hybridization. For transgenic lines, describe the transformation method, the number of independent lines analyzed and the generation upon which experiments were performed. For gene-edited lines, describe the editor used, the endogenous sequence targeted for editing, the targeting guide RNA sequence (if applicable) and how the editor was applied.*
Authentication	*Describe any authentication procedures for each seed stock used or novel genotype generated. Describe any experiments used to assess the effect of a mutation and, where applicable, how potential secondary effects (e.g. second site T-DNA insertions, mosiacism, off-target gene editing) were examined.*
